# Electric field-driven interfacial reduction of metal ions in microdroplets: gold, silver, and nickel†

**DOI:** 10.1039/d5sc04995d

**Published:** 2025-07-29

**Authors:** Sandeep Bose, Richard N. Zare

**Affiliations:** a Department of Chemistry, Stanford University CA 94305 USA zare@stanford.edu

## Abstract

A bulk aqueous solution containing 100 μM HAuCl_4_ has been shown to spontaneously form gold nanoparticles (Au NPs) in 2–3 days when stored at room temperature. We demonstrate that Au NPs can be spontaneously formed within a few microseconds to milliseconds when the same solution is sprayed in the form of microdroplets (10–30 μm in diameter) using N_2_ as the nebulizing gas under ambient conditions. The rapid formation of Au NPs establishes that the air–water interface of microdroplets plays a dominant role. The reduction of metal ions in water microdroplets is driven by electron transfer at the air–water interface of water microdroplets aided by the strong electric field and the lack of three-dimensional solvation at the surface. The reduction of metal is accompanied by the formation of H_2_O_2_ resulting in part from the recombination of OH˙ produced at the interface. We observed that the size of the Au NPs increases when the distance between the tip and collector increases suggesting the rapid nucleation and growth of Au NPs within the microdroplets. The nanoparticle generation in microdroplets is not limited to Au, and we extend the scope of this method to other metals such as silver (Ag) and nickel (Ni) indicating a minimal role of the metal's position in the electrochemical series. When polar protic solvents such as CH_3_OH, and C_2_H_5_OH replace water as a solvent, Au NPs are seen to be formed but at a much slower rate whereas in acetonitrile (ACN), the Au NPs' formation is negligible.

## Abstract 2

A bulk aqueous solution containing 100 μM HAuCl_4_ has been shown to spontaneously form gold nanoparticles (Au NPs) in 2–3 days when stored at room temperature.

## Graphical abstract

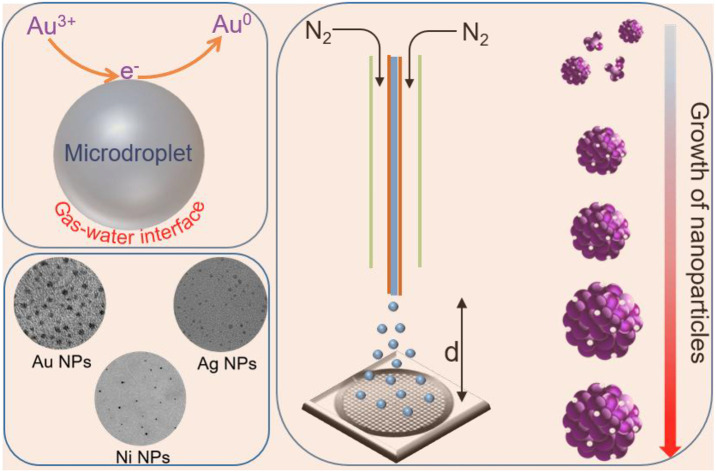

## Introduction

While bulk water is generally regarded as stable and chemically inert, its properties change significantly at the gas–water interface, especially in micron-sized droplets. At this interface, a strong electric field up to the order of 10^9^ V m^−1^ is generated.^1,2^ This strong electric field results from the uneven distribution of hydronium (H_3_O^+^) and hydroxide (OH^−^) ions across the droplet surface.^3^ This behavior makes the microdroplet interface reactive, facilitating redox reactions at the surface of a water microdroplet. Additional factors such as partial solvation,^4,5^ molecular orientation at the interface,^6,7^ local pH,^3^ and evaporation dynamics^8^ further enhance the redox activity in this confined environment. Previous reports showed that these redox properties at the gas–water interface can accelerate reactions and form unexpected products.^9–27^

Taking advantage of this, our group has previously shown that gold nanoparticles (Au NPs) can be spontaneously generated from the precursor HAuCl_4_ without any external reducing agent.^28^ Even the generated nanoparticles were shown to assemble in a template-free environment resulting in nanowire formation. A recent report demonstrated Au NPs can be produced spontaneously in 2–3 days when an aqueous solution of the precursor HAuCl_4_ is stored at room temperature.^29^ This report also suggests no growth of Au NPs occurs in microdroplets. This study challenges this later statement.

In this study, we revisit the formation of gold nanoparticles (Au NPs) in microdroplets to address several previously raised questions. Specifically, we aim to investigate the following:

(a) Does the nanoparticle formation occur within microdroplets, or is it restricted to the bulk phase?

(b) Does the growth of nanoparticles take place inside microdroplets?

(c) Can microdroplets be used as a general platform for synthesizing various metallic nanoparticles from their precursors, and does the metal's position in the electrochemical series significantly influence nanoparticle formation?

(d) What is the effect of different solvent environments – specifically polar protic solvents (CH_3_OH, C_2_H_5_OH) *versus* an aprotic solvent (ACN) – on the formation of Au NPs? Additionally, is the efficiency of Au NPs formation in organic solvents superior to that in H_2_O?

To demonstrate the formation of Au NPs in microdroplets is not limited to a specific droplet generation method, we utilized both pneumatic spraying and ultrasonic mesh nebulization techniques. Through a series of systematic experiments, we provide strong evidence supporting nanoparticle formation within microdroplets. Our results are critically analyzed in the context of prior findings, and we discuss points of agreement as well as divergence.

## Experimental section

**Fig. 1:**
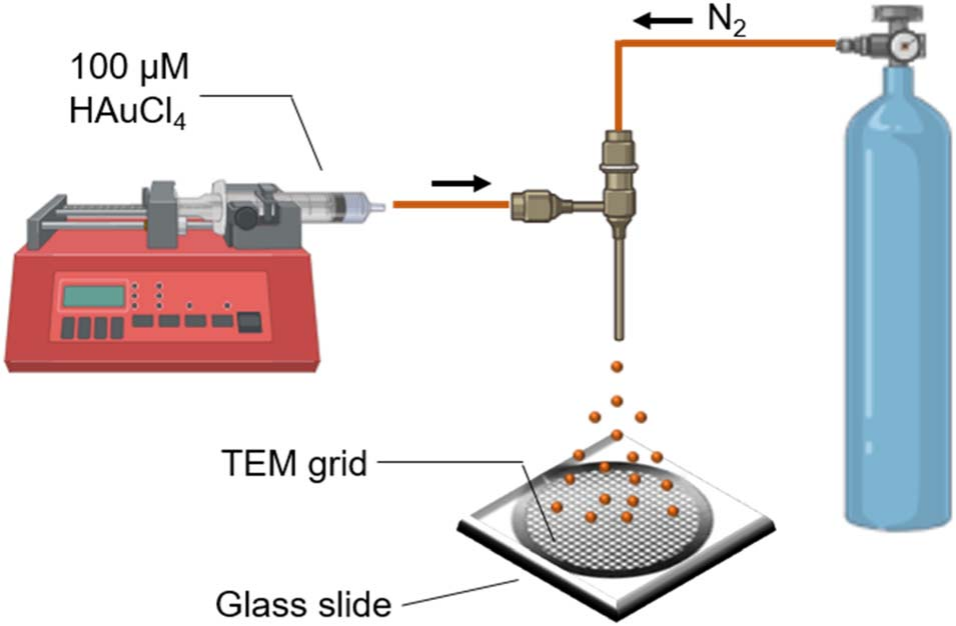
Schematic of the experimental setup utilized for the synthesis of Au NPs.

Fig. 1 displays the schematic of the pneumatic spray setup. A 1 mL syringe containing 100 μM of aqueous HAuCl_4_ was placed on top of a syringe pump (Harvard Apparatus). The precursor solution was pushed through an inner silica capillary (i.d. 50 μm) at a flow rate of 50 μL min^−1^. For generating microdroplets, N_2_ (120 psi) was used as a nebulizing gas. Microdroplets were collected directly on a transmission electron microscopy (TEM) grid after 30 s of spraying. The grid was then placed inside a desiccator for 5 min for further drying before it was taken for TEM measurement. For UV-Vis absorption studies, microdroplets were collected for 1 h. In a separate approach using an ultrasonic mesh nebulizer as shown in Fig. S1,† the same 100 μM aqueous HAuCl_4_ solution was used as the precursor, and microdroplets were similarly collected on a TEM grid for size measurement and in a vial for optical absorption studies.

## Results and discussion

**Fig. 2:**
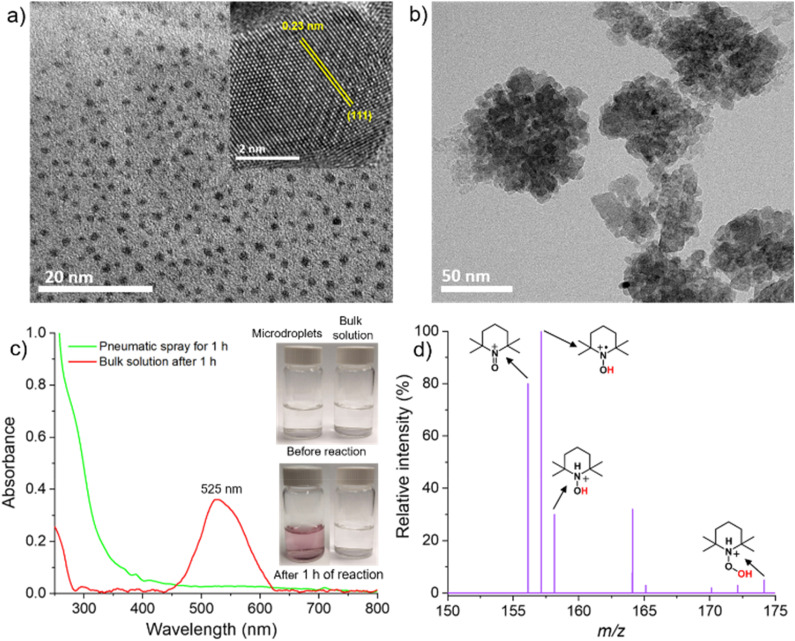
(a) TEM image of the Au NPs obtained after the spray. The inset shows a lattice distance of 0.23 nm corresponding to (111) of Au. (b) TEM image of the freshly prepared bulk HAuCl_4_ solution. (c) Comparison of UV-Vis absorption spectrum of the 1 h spray solution with the bulk solution. The inset shows a color change (formation of Au NPs) in the sprayed solution after 1 h, which is missing in the bulk solution. (d) TEMPO-based radical capture experiment that confirms the generation of H˙ and OH˙ during the spray.

When the microdroplets were collected on a TEM grid after 30 s and TEM was measured, we observed the formation of Au NPs (Fig. 2a). This figure depicts a high population of nanoparticles formed during the spray. The inset in Fig. 2a shows a lattice spacing of 0.23 nm corresponding to the (111) plane of Au.^28^ Additionally, when microdroplets were collected for 1 h and the UV-Vis absorption spectrum was recorded, we observed a feature at 525 nm (Fig. 2c), which arises from the plasmonic nature of Au NPs.^30^ When freshly prepared bulk HAuCl_4_ solution was drop-cast for TEM analysis after 30 s of its preparation, we did not observe any Au NPs (Fig. 2b). Also, no plasmonic feature of Au NPs was found in the absorption spectrum (Fig. 2c). The inset in Fig. 2c shows a color change of the sprayed solution after 1 h of spray caused by the formation of Au NPs, which is missing in the bulk solution. All these results confirm that Au NPs are formed in microdroplets on a very short time scale.

We also maintained the HAuCl_4_ precursor solution at room temperature for 2–3 days to check the formation of Au NPs as reported in previous literature.^29^ We noticed after 3 days, the solution color changed from colorless to light pink, indicating the formation of Au NPs. The color of the Au NPs prepared from bulk solution after 3 days closely matches that of the Au NPs prepared by 1 h of spraying (Fig. S2†). This is another indication that Au NPs are formed in the microdroplets and at a faster rate than that of the bulk.

At the air–water interface strong electric field and partial solvation led to the generation of reactive H˙ and OH˙, which has been confirmed by electron spin resonance spectroscopy.^31^ These radicals are more reactive than the H^+^ and OH^−^ and responsible for the redox reaction occurring at the interface. Thus, we believe the rapid reduction of Au ions at the interface is driven by electron transfer at the interface. We propose the following mechanism:

1 H_2_O ⇌ H^+^ + OH^−^

At the air–water interface:

2 OH^−^ → OH˙ + e^−^

3 H^+^ + OH^−^ ⇌ H˙ + OH˙

4 Au^3+^ + 3H˙ → Au + 3H^+^

5 Au^3+^ + 3e^−^ → Au

6 OH˙ + OH˙ → H_2_O_2_

Our proposed reaction mechanism varies from what Mishra and co-workers have proposed which shows the redox reaction in bulk solution requires H^+^ and OH^−^.^29^ First of all, bulk water is weakly ionized, *i.e.*, the H^+^ and OH^−^ concentrations are very low. Secondly, the ions in the bulk solution are heavily solvated. For, the reaction to take place in the bulk solution, the ions must overcome the solvation barrier, which is difficult unless an external mechanical force (stirring) or heat is applied. This explains the slow reduction of Au ions in the bulk phase that led to the formation of Au NPs in 2–3 days. Our group in 2018 proposed a similar mechanism for the reduction of Au ions unaware of the generation of H˙ and OH˙ at the interface.^28^ Later in 2019, we proposed the generation of radicals at the interface by detecting H_2_O_2_.^32^ The proposal was supported by Colussi who showed that electron transfer (ET) between surface-bound ions (OH^−^ + H^+^) at a water microdroplet interface can generate H˙ and OH˙, which promotes redox reaction at the interface.^33^ The same conclusion was also reached by Skurski and Simons.^34^ Our group has also published several reports where spontaneous reduction/oxidation by the H·˙/OH˙ is observed to occur at microdroplet interfaces.^35,36^ This led us to believe that the reduction of Au ions in the microdroplet is driven by electron transfer at and near the droplet interface and explains why the formation of Au NPs in microdroplets is generally fast.

The generation of H˙ and OH˙ during the spray was confirmed by capturing these radical species using TEMPO ((2,2,6,6-tetramethylpiperidin-1-yl)oxyl). TEMPO (5 mM) was introduced into the aqueous solution and the resulting solution was sprayed into the mass spectrum to detect the TEMPO-captured reactive species (Fig. 2d). The detection of a signal at *m*/*z* 157.1467, corresponding to TEMPO-H, along with a peak at *m*/*z* 158.1473 (TEMPO-H-H), provided evidence for the formation of H˙. Additionally, the signal at *m*/*z* 174.1494 (TEMPO-H-OH) confirmed the generation of OH˙ during spray.

From the above experiments, we conclude that Au NPs production in bulk is *spontaneous but slow*, whereas the same in microdroplets is *spontaneous and fast*.

We investigated whether any growth of Au NPs occurs in microdroplets. It is important to clarify that when we refer to nanoparticle growth within microdroplets, we specifically mean growth occurring during the spraying process, before deposition. Monitoring the nanoparticles after collecting the sprayed sample into a bulk solution does not accurately reflect the processes occurring within the microdroplets. Such an approach primarily captures post-spray growth dynamics in the solution phase, which closely resembles bulk solution behavior and therefore fails to provide direct insight into the unique environment and kinetics present within microdroplets during spraying.

**Fig. 3:**
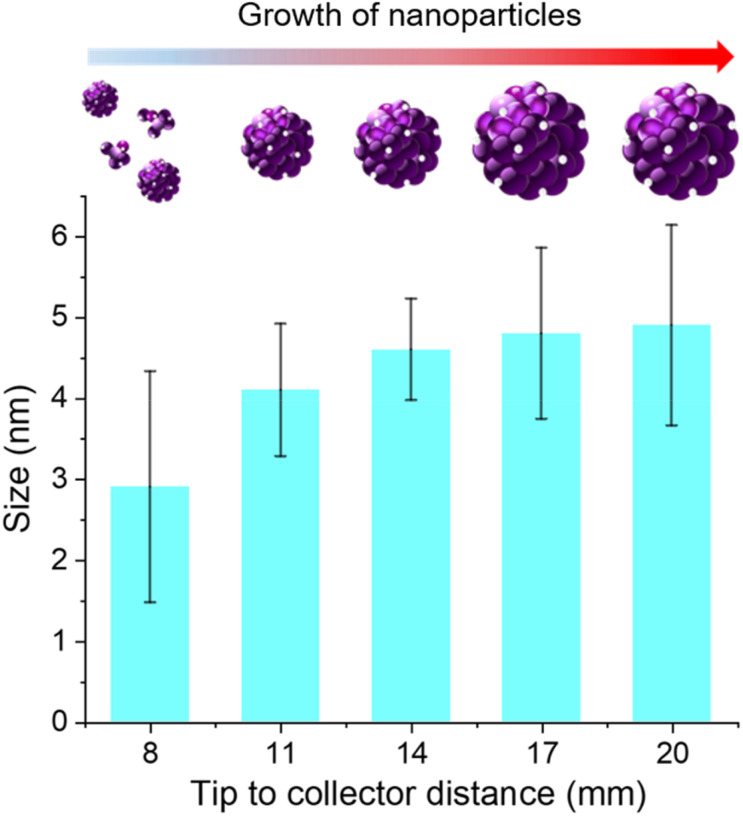
Average size of the Au NPs with the change in tip-to-collector distance.

We varied the tip-to-collector distance during spraying and the microdroplets were collected directly on top of a TEM grid after 30 s of spray. Finally, the TEM image was recorded to check the size of the nanoparticles formed. The TEM images at different tip-to-collector distances were shown in Fig. S3.† When the tip-to-collector distance increases, we notice an increase in the average size of the droplets (Fig. 3). Since the microdroplets were collected for only 30 seconds and subsequently placed in a desiccator for 5 minutes to ensure complete drying of the TEM grid, the time available for nanoparticle growth in the bulk solution phase was negligible. Therefore, any observed increase in the average nanoparticle size can be attributed to growth occurring within the microdroplets during the spraying process. This suggests that the confined environment of the microdroplets is facilitating or accelerating nanoparticle growth. We have also tested the nanoparticle growth using ultrasonic mesh nebulizers (Fig. S4†), and we observed similar results confirming that the accelerated growth is not specific to a particular spraying technique.

**Fig. 4:**
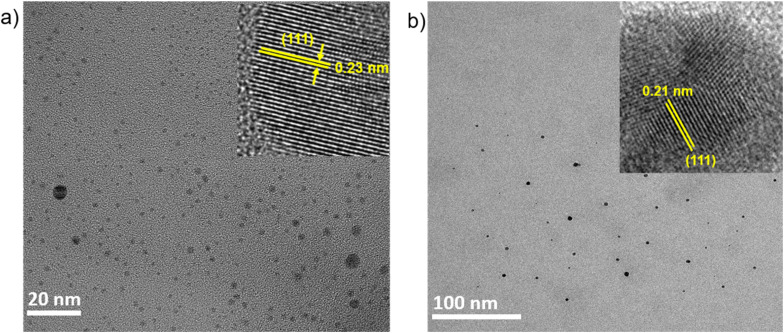
TEM images: (a) Ag NPs; and (b) Ni NPs formed after pneumatic spraying.

To investigate whether the spontaneous reduction is specific to Au ions or a more general phenomenon, we extended our study to other metal ions, namely Ag^+^ and Ni^2+^. For this purpose, aqueous solutions of 100 μM silver nitrate (AgNO_3_) and 100 μM nickel acetate tetrahydrate (Ni(OAc)_2_·4H_2_O) were prepared and subjected to pneumatic spraying. After spraying, the samples were collected and drop-casted for TEM. The TEM images show the formation of both the Ag NPs (Fig. 4a) and Ni NPs (Fig. 4b). The inset in Fig. 4a displays a lattice spacing of 0.23 nm corresponding to the (111) plane of Ag.^37^ Similarly, the inset of Fig. 4b depicts a lattice spacing of 0.21 nm corresponding to the (111) plane of Ni.^38^

We selected silver (Ag) and nickel (Ni) as model systems due to their significantly different standard reduction potentials. The Ag^+^/Ag redox couple has a standard reduction potential of +0.80 V, indicating that Ag^+^ ions are thermodynamically favorable for reduction in aqueous environments. In contrast, the Ni^2+^/Ni couple has a much lower standard reduction potential of – 0.25 V, suggesting that Ni^2+^ ions are less likely to undergo spontaneous reduction in water.

The reduction potential of this reaction, O_2_(aq) + 2H_2_O(aq) + 4e^−^ → 4OH^−^(aq), is +0.40 V in a bulk aqueous solution. This might seem to imply that only metal ions with reduction potentials more positive than +0.40 V, such as Ag^+^, should undergo reduction in aqueous solution under normal conditions. As expected, Ag NPs readily formed under spraying conditions. Surprisingly, however, we also observed the formation of Ni NPs, despite the unfavorable redox potential of Ni^2+^ under bulk aqueous conditions. Prior studies suggest that electric fields on the order of 10^9^ V m^−1^ across a ∼5 Å interfacial region can generate interfacial potentials of up to ∼3 V.^39^ Such strong fields are sufficient not only to drive water electrolysis (1.23 V) but also to oxidize OH^−^ to generate OH˙ (2.72 V)^39^ and reduce H^+^ to H˙, thereby producing highly reactive radical species. Based on this understanding, we propose that the reduction of Ni^2+^ observed in our system is driven by the *in situ* generated potential at the air–water interface of the microdroplets. These findings suggest microdroplets can be used to synthesize a variety of metallic nanoparticles irrespective of their positions in the electrochemical series.

**Fig. 5:**
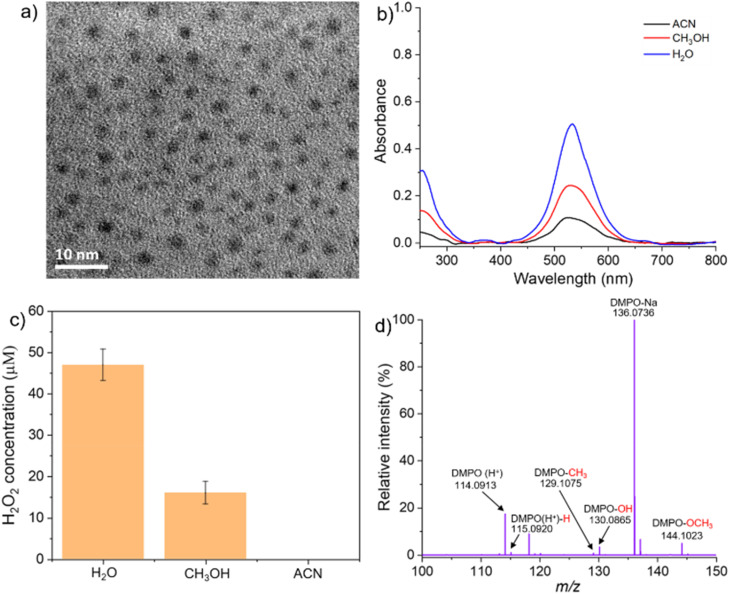
(a) TEM image of the Au NPs formed when CH_3_OH was used as a solvent. (b) UV-Vis absorption spectra of Au NPs in H_2_O, CH_3_OH, and ACN solvent. (c) Concentration of H_2_O_2_ generated using different solvents during the formation of Au NPs. (d) DMPO-based radical capture experiments to confirm the generation of ˙CH_3_ and ˙OCH_3_ during the formation of Au NPs using CH_3_OH as a solvent in microdroplets.

We also explored the influence of different solvents on the formation of Au NPs to determine whether nanoparticle synthesis is specific to aqueous media or can be extended to organic solvents. To this end, we selected polar protic solvents (CH_3_OH and C_2_H_5_OH) and a polar aprotic solvent acetonitrile (ACN) as alternatives to water. When anhydrous CH_3_OH (extra dry) was used as a solvent for spray, we observed the formation of Au NPs (Fig. 5a). The TEM image displays a fairly high population of nanoparticles formed during the spray. Similar observations were made when C_2_H_5_OH was used as a solvent (Fig. S5a†). This proves that Au NPs formation is not specific to water, and polar protic solvents also form Au NPs in microdroplets. When ACN was used as a solvent, the population of Au NPs was much less (Fig. S5b†).

As mentioned earlier, water produces H˙ and OH˙ at the interface of microdroplets, which helps in the reduction of metal ions. Because both CH_3_OH and C_2_H_5_OH contain polar O–H bonds, we believe, it undergoes cleavage at the interface to form H˙ and OH˙ as follows:

For methanol:

7 CH_3_OH → CH_3_O˙ + H˙

8 CH_3_OH → ˙CH_3_ + OH˙

9 OH˙ + OH˙ → H_2_O_2_

For ethanol:

10 C_2_H_5_OH → C_2_H_5_O˙ + H˙

11 C_2_H_5_OH → ˙C_2_H_5_ + OH˙

12 OH˙ + OH˙ → H_2_O_2_

The generated electrons, and H˙ radicals subsequently facilitate the reduction of Au ions to metallic Au as shown in eqn (4) and (5).

To validate our proposed mechanism, we employed 5,5-dimethyl-1-pyrroline *N*-oxide (DMPO) as a spin-trapping agent to capture radical species generated during the spray process using CH_3_OH as the solvent. DMPO was added to the alcoholic precursor solution and the mixture was directly introduced into the mass spectrometer. The detection of DMPO-adduct peaks at *m*/*z* 115.0920 (DMPO(H^+^)-H), 129.1075 (DMPO-CH_3_), 130.0865 (DMPO-OH), and 144.1023 (DMPO-OCH_3_), corresponding to H˙, OH˙, ˙CH_3_, and ˙OCH_3_ respectively (Fig. 5b), confirms the generation of these species during the spray. This evidence supports our proposed radical-based reduction pathways of Au ions in CH_3_OH-containing microdroplets. Although ACN lacks polarizable H or O atoms, a small amount of Au NPs formation was still observed. This is likely from trace amounts of water present as an impurity in the ACN or to the interaction of ACN microdroplets with atmospheric moisture during the spray.

Among all the solvents tested, water was found to be the most effective medium for the reduction of Au ions and the formation of Au NPs. This result is opposite to the findings previously reported by Mishra and co-workers, where CH_3_OH was shown to be more effective than water.^29^ To support our observation, we compared the amount of H_2_O_2_ generated during nanoparticle formation in each solvent, based on the rationale that greater metal ion reduction correlates with increased H_2_O_2_ production. Our results showed that H_2_O_2_ formation was highest when water was used as the solvent, followed by CH_3_OH, while no detectable H_2_O_2_ was observed in the presence of ACN (Fig. 5c). Furthermore, UV-Vis absorption measurements revealed the highest intensity of the characteristic Au NPs surface plasmon resonance peak at 525 nm for water, followed by CH_3_OH, and then ACN (Fig. 5d). Importantly, identical concentrations of HAuCl_4_ were used across all experiments to ensure comparability. These findings indicate that water facilitates the reduction of Au ions more efficiently than either polar protic or aprotic organic solvents.

## Conclusions

This study demonstrates that Au NPs are formed in water microdroplets and the process is both spontaneous and rapid, in contrast to the significantly slower, though also spontaneous, process observed in bulk aqueous solution, which requires several days. Additionally, our experiments confirm that nanoparticle growth occurs within the microdroplets during the spray process. The high interfacial electric field and partial solvation at the air–water interface promote the generation of reactive H˙ and OH˙, which drive the reduction of Au^3+^ to Au^0^ and the formation of H_2_O_2_ as the oxidation product. Using TEMPO as a radical scavenger, we confirmed the presence of these reactive species during spraying. Au NPs formation was observed in CH_3_OH, and C_2_H_5_OH but was negligible in ACN, likely caused by the absence of polarizable H or O for radical generation. Among all solvents tested, water proved to be the most efficient, showing the highest levels of H_2_O_2_ and UV-Vis absorbance at 525 nm. Furthermore, we extended the method to the production of Ag NPs and Ni NPs, demonstrating the reduction of metal ions regardless of their standard redox potentials, highlighting the unique redox environment of microdroplets. These findings establish microdroplets as a powerful platform for rapid, and green synthesis of metal nanoparticles across a range of metals and solvent systems.

## Author contributions

S. B. performed most of the experiments, analysis, and drafting of the first version of the manuscript. R. N. Z. proposed this project and revised the manuscript.

## Conflicts of interest

There are no conflicts to declare.

## Supplementary Material

SC-OLF-D5SC04995D-s001

## Data Availability

The data supporting this article have been included as part of the ESI.†
