# Supplementary material for: Electric field-driven interfacial reduction of metal ions in microdroplets: gold, silver, and nickel
Source: Chem Sci. 2025 Jul 29;16(34):15455–61. doi: 10.1039/d5sc04995d (PMC12305125; doi:10.1039/d5sc04995d)
Supplement: SC-016-D5SC04995D-s001 [file SC-016-D5SC04995D-s001.pdf]

Supplementary Information (SI) for Chemical Science.
This journal is © The Royal Society of Chemistry 2025

Supporting Information

Electric Field–Driven Interfacial Reduction of Metal Ions in Microdroplets: Gold, Silver, and Nickel

Sandeep Bose, Richard N.Zare*

*Department of Chemistry, Stanford University, CA 94305 United States

Email: zare@stanford.edu

Table of Contents

Items	Description	Page No.
	Chemicals required	S1
	Instrumentation	S1
	H_2O_2 quantification	S2
Figure S1	Photographic image of the ultrasonic mesh spray device	S2
Figure S2	Comparison of the color of Au NPs formed in microdroplets after 1 hour with those prepared in bulk solution over 3 days	S2
Figure S3	TEM image of the Au NPs obtained at different tip-to-collector distance using pneumatic spray	S3
Figure S4	Plot of the average size of the Au NPs against the tip-to-collector distance (ultrasonic spray)	S4
Figure S5	TEM images of the Au NPs formed in presence of C_2H_5OH and ACN	S4

Chemicals required

Chloroauric acid ($HAuCl_4.4H_2O$) was purchased from Sigma Aldrich. Silver nitrate ($AgNO_3$) and nickel acetate [$Ni(OAc)_2.4H_2O$] was obtained from Fischer Scientific. 2,2,6,6-tetramethylpiperidin-1-yl)oxyl (TEMPO) and 5,5-dimethyl-1-pyrroline N-oxide (DMPO) were purchased from Sigma Aldrich. All the solvents were purchased from Fischer Scientific. All the solvents and chemicals were used as purchased without further purification.

Instrumentation

The UV-Vis absorption studies were conducted using CLARIOstar, BMG LABTECH UV-Vis spectrometer. The transmission electron microscopy (TEM) measurements were performed using FEI Titan environmental TEM with high brightness field emission gun (X-FEG). A high-resolution LTQ Orbitrap mass spectrometer (Thermo Fisher Scientific, San Jose, CA, USA) was utilized to accurately determine the exact *m/z* values of the target ions, operating at a resolution of 60,000.

H_2O_2 quantification

For the quantification of H_2O_2, a colorimetric method based on the oxidation of iodide (I^-) to triiodide (I_3^-) was employed. In this procedure, 20 µL each of two reagent solutions - Solution A (containing 0.4 M KI, 0.1 M NaOH, and 0.02 mM ammonium molybdate) and Solution B (0.1 M potassium hydrogen phthalate) were combined with 20 µL of the sample. The iodide ion (I^-), which does not exhibit a characteristic absorption peak, is catalytically oxidized to I_3^- in the presence of ammonium molybdate and hydrogen peroxide. The resulting I_3^- ion displays a distinct absorbance at 353 nm. The mixture was analyzed using a UV-vis spectrophotometer by monitoring the absorbance at 353 nm. A calibration curve was generated using standard H_2O_2 solutions. Each experiment was performed in triplicate to ensure reproducibility. The reported values represent the mean of the three independent measurements, and the corresponding data were plotted accordingly. Error bars in the graph indicate the standard error, which reflects the variability of the measurements relative to the mean. The standard error (SE) was determined using the formula $SE = \sigma/\sqrt{n}$, where σ denotes the standard deviation and n is the number of replicates.

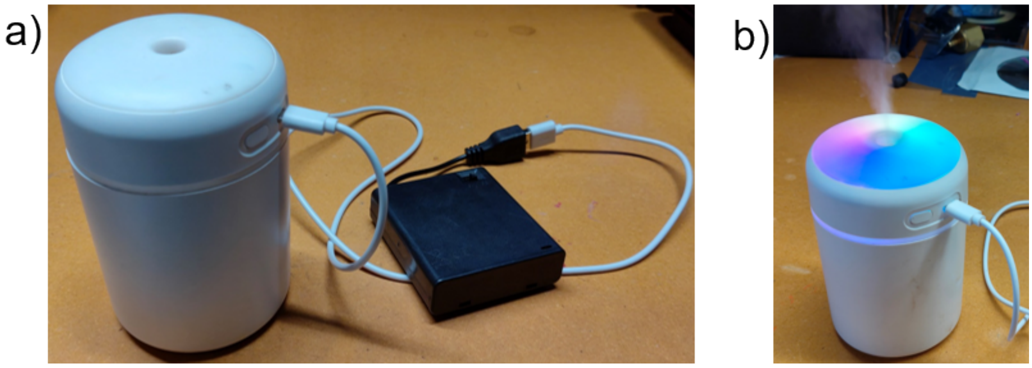

Fig. S1 a) Photographic image of the commercially available ultrasonic mesh spray device used for Au NPs generation. b) Image of the spray plume coming out of the ultrasonic mesh spray device.

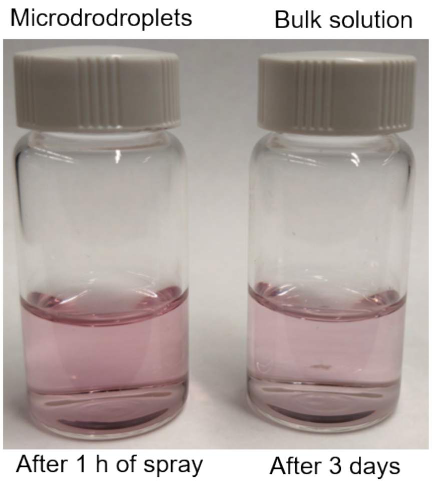

Fig. S2 Comparison of the color of Au NPs formed using microdroplets (after 1 h) with that of the bulk solution (after 3 days).

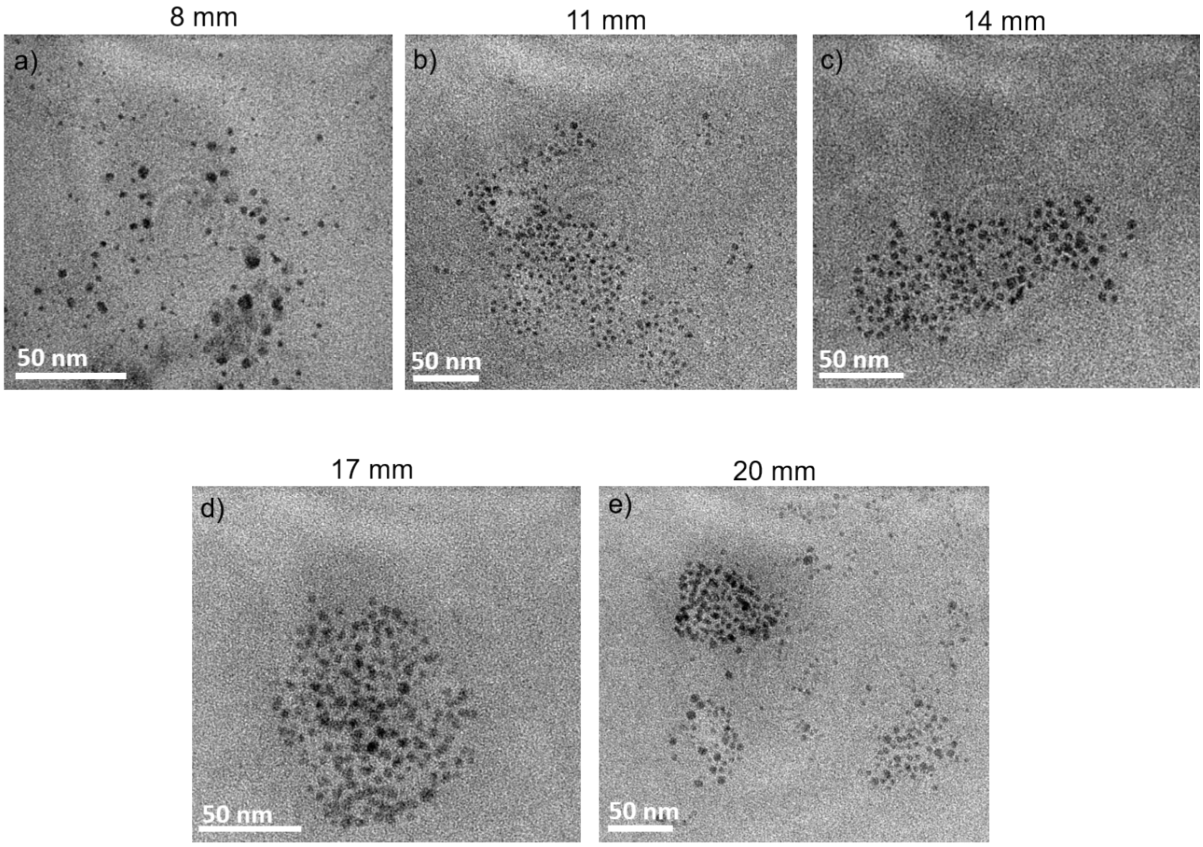

Fig. S3. TEM image of the Au NPs obtained at different tip-to-collector distance using pneumatic spray shown in Fig. 3.

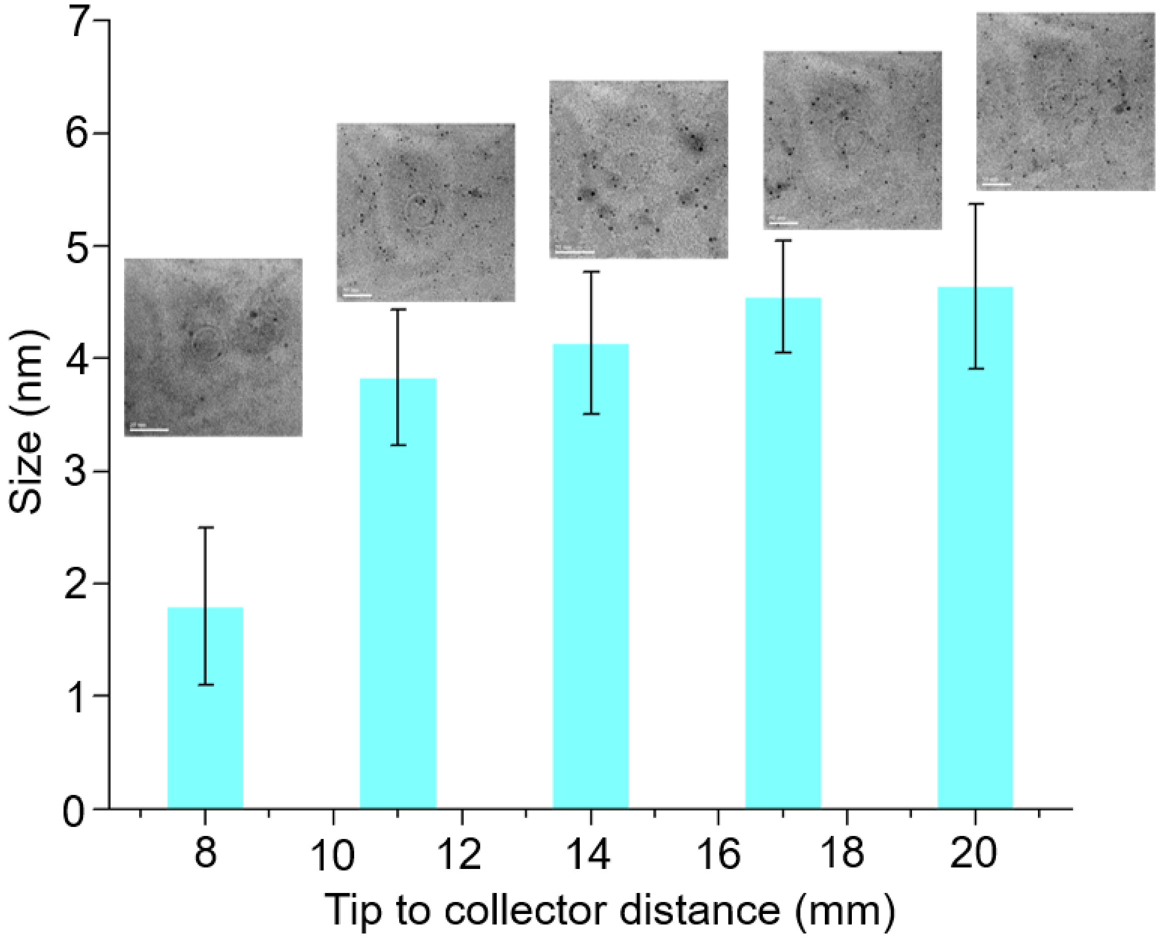

Fig. S4. A plot of the average size of the Au NPs against the tip-to-collector distance using ultrasonic mesh nebulizer. The inset shows the corresponding images from where the average size is calculated.

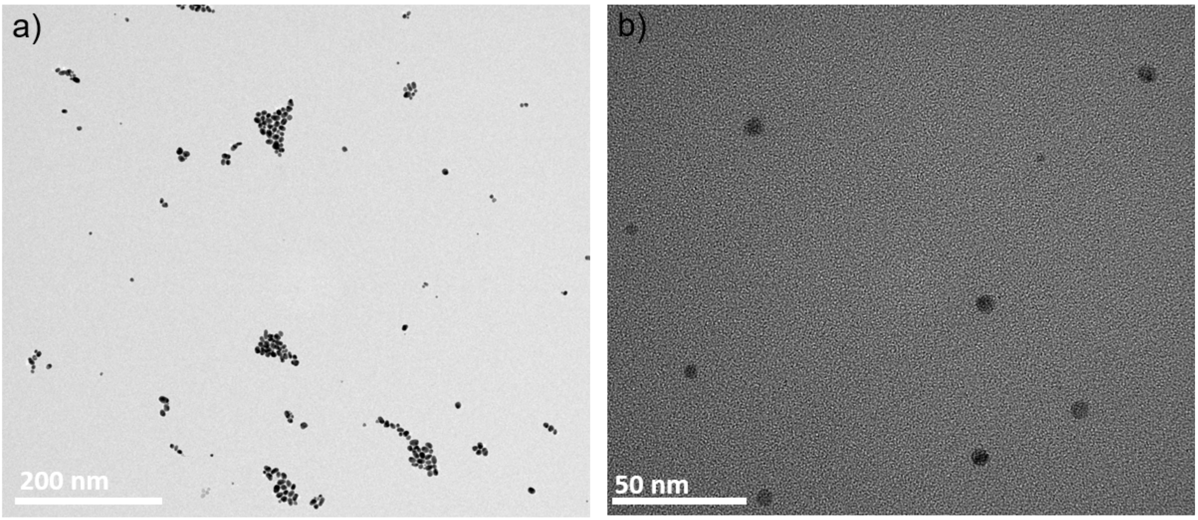

Fig. S5. a) TEM image of the Au NPs obtained when C_2H_5OH was used as a solvent. b) TEM image of the Au NPs obtained when ACN was used as a solvent.
